# Supplementary material for: Transcriptome Analysis Reveals Critical Genes Involved in the Response of Stropharia rugosoannulata to High Temperature and Drought Stress
Source: Curr Issues Mol Biol. 2025 Oct 10;47(10):835. doi: 10.3390/cimb47100835 (PMC12563022; doi:10.3390/cimb47100835)
Supplement: Supplementary file 1 [file cimb-47-00835-s001.zip › Table S1 Primer for qRT-PCR of eight candidate genes.pdf]

Table S1. Primer for qRT-PCR of eight candidate genes.

Primer name	Forword primer	Reverse primer
GAPDH	CACGGCCACTGGAAGCA	TCCTCAGGGTTCCTGATGCC
TRINITY_DN164_c0_g1 (*ATF2*)	GCGGCAAGGCGGACAAATAA	GAGATAAGGGCGGATGTGAGACG
TRINITY_DN351_c0_g1 (*PTP2_3*)	GGAGGGTTTATTGCTGTAGATG	GATATGGAAGGCGTTGTTGG
TRINITY_DN3855_c0_g1 (*BCK1*)	TCGGCGGACGACTACTCTAT	TTCACCCTTCTCGAAACCTG
TRINITY_DN5930_c0_g1 (*SHO1*)	AGAAAGCAGAGCGACTAACGG	CCACAAGATACGGACGAGACC
TRINITY_DN6367_c0_g1 (*GPCR*)	GCAGACCGATCCTACATACC	AAAGGAACTTGGAGGAGATG
TRINITY_DN822_c1_g1 (*SAC7*)	AAATGCCACCGAAATACAGG	CGGCGAATGAATGACTTGTA
TRINITY_DN4007_c0_g1 (*GPA1*)	AGCCCGATGATAACGACCCA	GGATGACAGCCCTCCACGAC
TRINITY_DN2810_c0_g1 (*PRMT5*)	TGTGCCAGCCTTGCTACTTT	CCAGGATTGAGCCTCCATAA
