# Supplementary material for: Transcriptome Analysis Reveals Critical Genes Involved in the Response of Stropharia rugosoannulata to High Temperature and Drought Stress
Source: Curr Issues Mol Biol. 2025 Oct 10;47(10):835. doi: 10.3390/cimb47100835 (PMC12563022; doi:10.3390/cimb47100835)
Supplement: Supplementary file 1 [file cimb-47-00835-s001.zip › Table S2 The distribution of transcript assembly length.pdf]

Table S2. The distribution of transcript assembly length.

Mean length (bp)	GC (%)	N50	Total transcipts	Total genes	Total assembled bases(bp)
3,002	49.74	4,550	74,571	16,233	223,923,640
