# Supplementary material for: Transcriptome Analysis Reveals Critical Genes Involved in the Response of Stropharia rugosoannulata to High Temperature and Drought Stress
Source: Curr Issues Mol Biol. 2025 Oct 10;47(10):835. doi: 10.3390/cimb47100835 (PMC12563022; doi:10.3390/cimb47100835)
Supplement: Supplementary file 1 [file cimb-47-00835-s001.zip › Table S3 Statistics of function annotation results.pdf]

Table S3. Statistics of function annotation results.

Items	Database	Number	Percentage (%)
-	KOG	4,709	30.21
-	TF	352	2.26
-	KEGG	3,039	19.49
-	GO	3,728	23.91
-	NR	8,591	55.11
-	Pfam	8,550	54.85
Total Coding Sequence	-	15,589	100
Annotated	-	10,248	65.74
Un-notated	-	5,341	34.26
