# Supplementary figures and images for: Transcriptome Analysis Reveals Critical Genes Involved in the Response of Stropharia rugosoannulata to High Temperature and Drought Stress
Source: Curr Issues Mol Biol. 2025 Oct 10;47(10):835. doi: 10.3390/cimb47100835 (PMC12563022; doi:10.3390/cimb47100835)

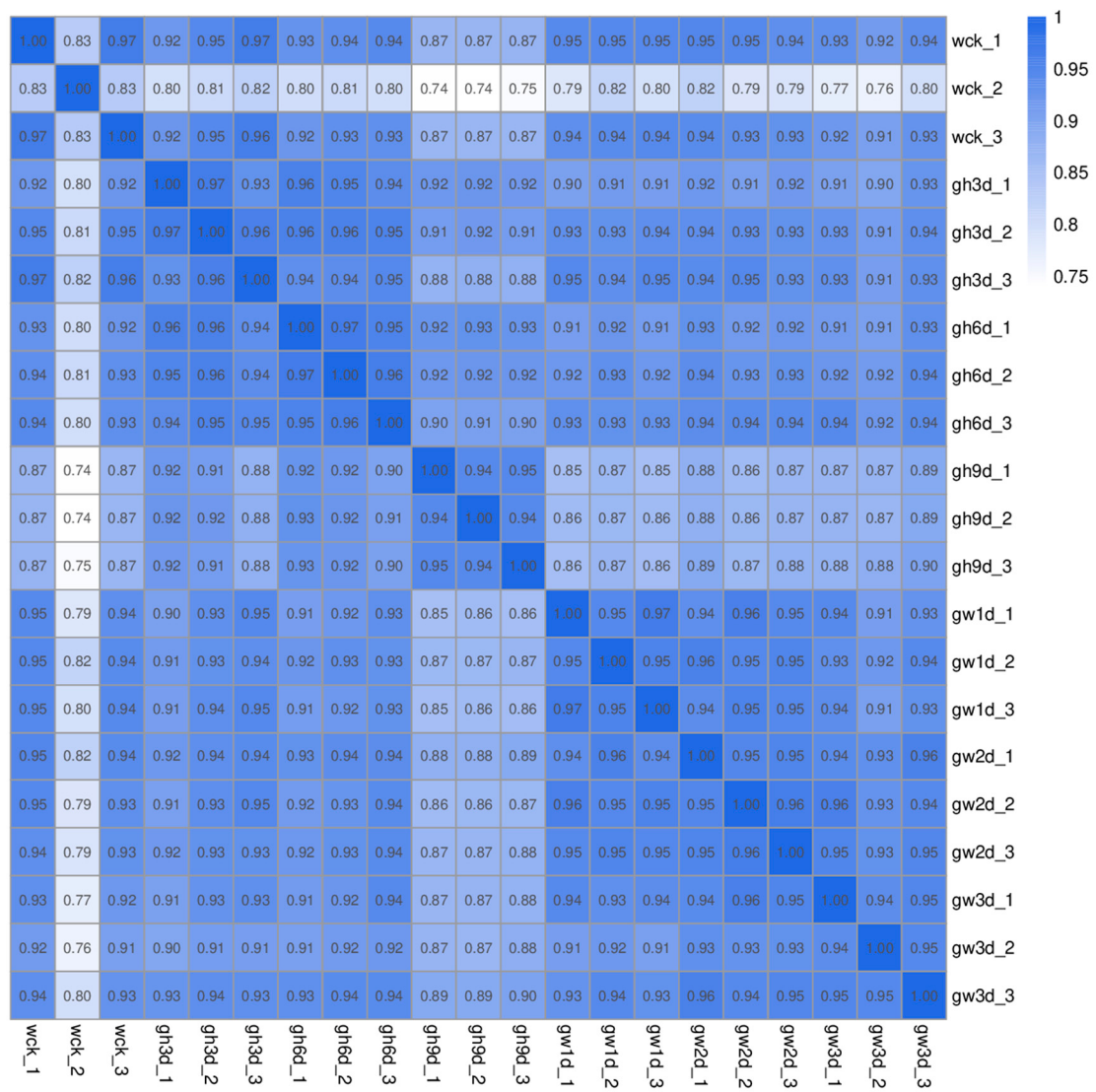

**Figure S1.** Correlation heatmap of all samples.

Supplement: Supplementary file 1 [file cimb-47-00835-s001.zip › Figure S1 Correlation heatmap of all samples.pdf]
